# Generation of Monoclonal Antibodies against Highly Conserved Antigens

**DOI:** 10.1371/journal.pone.0006087

**Published:** 2009-06-30

**Authors:** Hongzhe Zhou, Yunbo Wang, Wei Wang, Junying Jia, Yuan Li, Qiyu Wang, Yanfang Wu, Jie Tang

**Affiliations:** Center of Infection and Immunity, National Key Laboratory of Biomacromolecules, Institute of Biophysics, Chinese Academy of Sciences, Beijing, P.R.China; New York University School of Medicine, United States of America

## Abstract

**Background:**

Therapeutic antibody development is one of the fastest growing areas of the pharmaceutical industry. Generating high-quality monoclonal antibodies against a given therapeutic target is very crucial for the success of the drug development. However, due to immune tolerance, some proteins that are highly conserved between mice and humans are not very immunogenic in mice, making it difficult to generate antibodies using a conventional approach.

**Methodology/Principal Findings:**

In this report, the impaired immune tolerance of NZB/W mice was exploited to generate monoclonal antibodies against highly conserved or self-antigens. Using two highly conserved human antigens (MIF and HMGB1) and one mouse self-antigen (TNF-alpha) as examples, we demonstrate here that multiple clones of high affinity, highly specific antibodies with desired biological activities can be generated, using the NZB/W mouse as the immunization host and a T cell-specific tag fused to a recombinant antigen to stimulate the immune system.

**Conclusions/Significance:**

We developed an efficient and universal method for generating surrogate or therapeutic antibodies against “difficult antigens” to facilitate the development of therapeutic antibodies.

## Introduction

Both *in vitro* and *in vivo* approaches are used for monoclonal antibody development. *In vitro* approaches, such as phage display or ribosomal display, select antibody sequences from an immunoglobulin variable chain cDNA library, while *in vivo* approaches use immunized animals as hosts and screen for monoclonal antibodies with conventional hybridoma techniques. Since the animal immune system is designed by nature for high affinity and highly-specific antibody development, the *in vivo* approach is obviously more cost effective than the *in vitro* approach.

Tolerance – the ability of the immune system to prevent responses to self antigens – makes it difficult to generate a strong immune response in mice with a mouse self-antigen or highly conserved human antigen [1]. Currently, specific knockout mice are used to overcome the immune tolerance associated with self-antigens. Generation of knockout mice for every mouse antigen that we need to raise antibodies for is obviously both costly and time-consuming. In certain cases when knockout mice are immune-deficient or die prematurely, it is even more difficult if not impossible to raise antibodies against those antigens. Systematic autoimmune diseases, however, indicate the presence of anergic self-reactive B and T cells in the immune repertoire, and present opportunities for the loss of tolerance leading to strong antibody responses against self antigens [2]. High titers of serum antibodies reacting to self-antigens are found in mouse human SLE-like models (NZB/W and MRL/lpr mice) without prior immunization with the corresponding self-antigens [3], [4]. In fact, auto-immune NZB mice have been used successfully to generate antibodies against carbohydrate determinants in myelin-associated glycoprotein [5], capsular polysaccharides in group B Neisseria meningitides [6], and glycosphingolipid asialo-GM1 [7]. Recently, monoclonal antibodies against the highly conserved bovine recombinant prion protein have also been generated using NZB/W mice [8]. However, due to the multi-specificity and low affinity of auto-antibodies from NZB/W mice, there are still doubts whether therapeutic antibodies with high affinity and high specificity, as well as the desired biological activities, can be obtained from this type of mouse.

In this report, three pro-inflammatory cytokines, TNF-alpha, MIF and HMGB1 were used as test antigens in our efforts to exploit a new method to generate antibodies against highly conserved antigens. All three have been implicated as good drug targets for inflammation related diseases [9], [10], [11]. Human MIF and HMGB1 are representatives of highly conserved proteins and mouse TNF-alpha represents mouse self antigens. Our results demonstrate that monoclonal antibodies with high affinity and high specificity can be generated from NZB/W mice and that some of these antibodies possess neutralizing activity which is very useful in target validation and therapeutic antibody development.

## Methods

### Ethics Statement

Maintenance of mice and experimental procedures were approved by the Animal Welfare and Research Ethics Committee of the Institute of Biophysics, Chinese Academy of Sciences.

### Recombinant protein expression

Human MIF and mouse TNF-alpha were cloned into a PET-24a vector (Novagen) and expressed in *Escherichia coli* (*E.coli*). MT-tagged HMGB1 was cloned into a PET-41d-MT vector (a pET41a variant with the GST coding sequence deleted and a sequence coding for the DQVHFQPLPPAVVKLSDAL polypeptide added to the C-terminus). The proteins were expressed in *E.coli* strain BL21(DE3) and purified by affinity chromatography using Ni-NTA His bind resins (Novagen) according to the manufacturer's instructions. All GST-tagged HMGB1 constructs were cloned into the expression vector pET41a, expressed in *E.coli* strain BL21(DE3) and purified with GSTBind Purification Kits (Novagen) according to the manufacturer's protocol.

### Immunization and hybridoma selection

Female BALB/c and NZB/W mice (12 weeks old) were injected subcutaneously with 50 µg of purified recombinant protein emulsified in complete Freund's adjuvant. Two additional injections of 50 µg of antigen emulsified in incomplete Freund's adjuvant were followed at bi-weekly intervals starting four weeks after the first immunization. Ten days after the second boost, the serum antibody titer was tested using ELISA. Two weeks after the second boost, the mice were given a final booster injection intraperitoneally with 50 µg protein. Three days after the last injection, spleen cells from the immunized mice were fused with myeloma Sp2/0 cells [12]. ELISA was employed for screening antigen-specific monoclonal antibodies.

### ELISA

Antigen (10 µg/ml) was coated on microtiter plates overnight at 4°C. 100 µl of antisera diluted in PBST was added and plates were incubated for 2 hours at room temperature (RT). After washing, a 1∶1000 dilution of HRP-conjugated goat anti-mouse Ig polyclonal antibody (R&D Systems) was added for 1 hour at RT. 100 µl substrate (TMB system) was added and plates were read at 450 nm. For dissociated constant (Kd) measurement, purified monoclonal antibodies were used as the primary antibody with a two-fold serial titration. The Kd was determined as the antibody concentration that can achieve 50% of the maximum ELISA reading. For antibody isotype characterization, HRP-conjugated rat anti-mouse IgG1, IgG2a or IgG2b monoclonal antibodies (BD Pharmingen) were used as the secondary antibody instead of the polyclonal antibody described above.

### Yeast display

Primers were designed for cloning the cDNA encoding human MIF. PCR-amplified fragments were gel-purified using a gel-extraction kit (Tiangen), and then transformed into EBY100 cells along with a Not I/EcoR I-digested-pYDS vector, according to a LiAC-based protocol as previously described [13]. Yeast clones expressing human MIF were cultured in SD-CAA media (2% (w/v) dextrose, 0.67% (w/v) yeast nitrogen base, and 1% (w/v) Casamino acids) for 36 hours at 30°C.

Approximately 4×10^8^ yeast cells were pelleted by centrifugation and transferred to SG-CAA (2% (w/v) galactose) media to induce protein expression and then cultured for 36 hours at 20°C. Induced yeast cells were pelleted and washed in PBS with 1% (w/v) BSA. Cells were incubated with the indicated concentrations of biotinylated or non-biotinylated anti-MIF antibodies for half an hour at room temperature. Cells were then washed twice with PBS-BSA and incubated with FITC-labeled goat anti-mouse Ig for 30 minutes. Finally, cells were washed twice with PBS-BSA and analyzed by flow cytometry.

### *In vitro* functional assays

Raw264.7 cells were treated with 3 µg/ml recombinant human MIF with or without anti-MIF antibody 10C3(titrated serially with a starting concentration of 100 µg/ml). After 16 hours, the supernatant was collected and nitric oxide concentration was measured with the Griess reagent system (Promega) according to the manufacturer's protocol.

8×10^5^ Raw264.7 cells were stimulated with 1 µg/ml HMGB1 for 8 hours, with or without 50 µg/ml anti-HMGB1 antibodies. Total mRNA was extracted with Trizol (Invitrogen) according to the manufacturer's protocol. 2 µg of total RNA was used in a 25 µl final volume oligo dT primed reverse transcription reaction using a MLV RT kit (Promega) according to the manufacturer's protocol. Target genes were assayed in 20 µl PCR reactions containing 10 µl SYBR Green PCR Mix (Qiagen), 5pmol sense/anti-sense primers and 2 µl of the RT reaction. 40 cycles of quantitative PCR was performed in a Rotor-Gene 6000 real time PCR machine (Corbett). Primers used in the IL-6 mRNA RT-PCR assay were as follows:

Mouse IL-6 primers for real-time PCR assay:

Sense: 5′-AACGATGATGCACTTGCAGA

Anti-sense: 5′-GAGCATTGGAAATTGGGGTA

Mouse beta-actin primers for real-time PCR assay:

Sense: 5′-GCTACAGCTTCACCACCACAG

Anti-sense: 5′-GGTCTTTACGGATGTCAACGTC

### LPS-induced sepsis model

6–8 week-old C57BL/6 mice, weighing between 18 and 20 g, were randomly divided into groups. Mice were injected intraperitoneally with indicated doses of Lipopolysaccharides (LPS) (from *E. coli* strain 0111:B4 , Sigma L2630) or LPS plus indicated doses of monoclonal antibodies. The number of surviving mice was recorded every six hours.

### Statistical Analysis

Data were expressed as the means±SEM. Differences were analyzed using Dunnett's multiple comparison tests. p values <0.05 were regarded as significant. Statistical significance of survival rate was determined using log-rank (Mantel-Cox) test.

## Results

**Figure 1:**
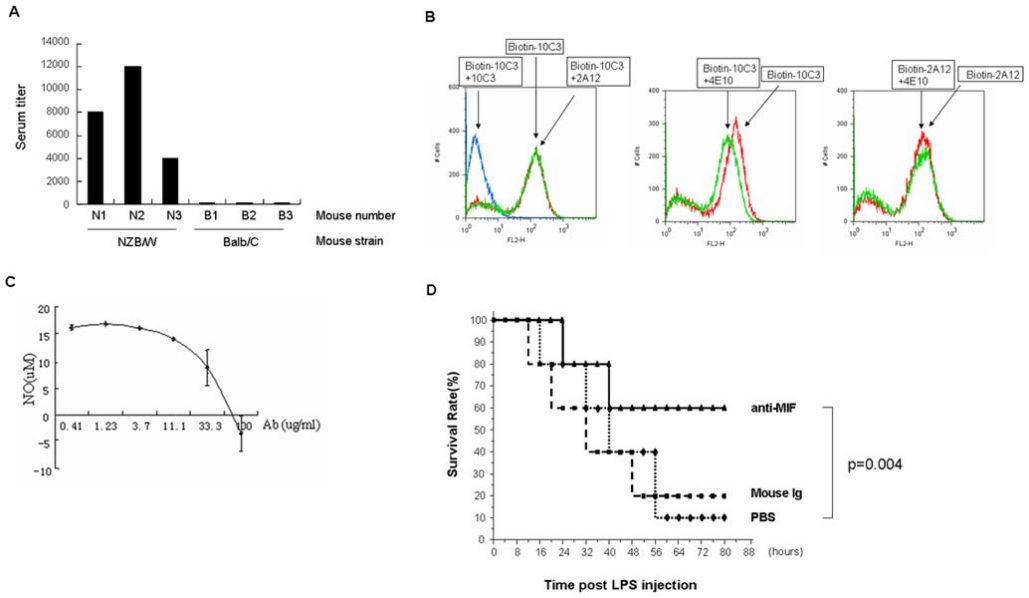
Generation of high affinity, biologically active anti-MIF antibodies from NZB/W mice. (A) Use of NZB/W mice to break immune tolerance.NZB/W or BALB/C mice were immunized with His tagged MIF protein. Serum titers were measured by ELISA using GST-MIF coated plates. Serum titer was defined as the highest dilution of serum at which the A450 ratio (A450 of sample/A450 of negative serum) was greater than 2.0. (B) Competition assays between three anti-MIF clones, 10C3, 2A12 and 4E10. Human MIF was displayed on the surface of yeast cells. Yeast cells were incubated with 10 nM of biotin-labeled antibodies, with or without 100 nM of non-biotinylated antibodies as competitors. Streptavidin-PE was used for secondary labeling. (C) 10C3 inhibits MIF-induced nitric oxide secretion in a macrophage cell line. Raw264.7 cells were stimulated with recombinant MIF (3 µg/ml) and increasing concentrations of anti-MIF antibody 10C3 for 16 hours. Nitric oxide concentration in the supernatant was detected using the Griess assay. (D) 10C3 protects mice from LPS-induced sepsis. 8 week C57BL/6 female mice, 10 mice/group, were injected intraperitoneally with 22.5 mg/kg LPS, along with anti-MIF antibody 10C3 or isotype control antibodies (5 mg/kg). The number of surviving mice was recorded every six hours.

Macrophage migration inhibitory factor (MIF) is an important mediator of inflammatory responses and a drug target for sepsis and auto-immune diseases [10], [14]. The human and mouse MIF proteins share 89% sequence identity. When NZB/W and BALB/C mice were immunized with a recombinant human MIF protein, only NZB/W mice generated good serum antibody titers (Fig. 1A). From the immunized NZB/W mice, 6 hybridoma clones (out of 136 clones screened) secreting antibodies specific to human MIF were obtained from NZB/W mice. Three of these, 4E10 (IgG2a), 10C3 (IgG2b) and 2A12 (IgG2b), are high-affinity antibody clones with dissociation constants (Kds) of 2.5 nM, 1.3 nM and 0.1 nM, respectively (measured by ELISA). In a competition assay, 10C3 and 4E10 did not compete with 2A12, while 4E10 partially competed with 10C3, suggesting that these three clones bind to different epitopes on MIF (Fig. 1B). Of the three clones, 10C3 blocked *in vitro* MIF-induced nitric oxide secretion in the macrophage cell line Raw264.7 (Fig. 1C). Furthermore, 10C3 showed *in vivo* activity when it rescued LPS-induced lethality in a mouse sepsis model (Fig. 1D).

**Figure 2:**
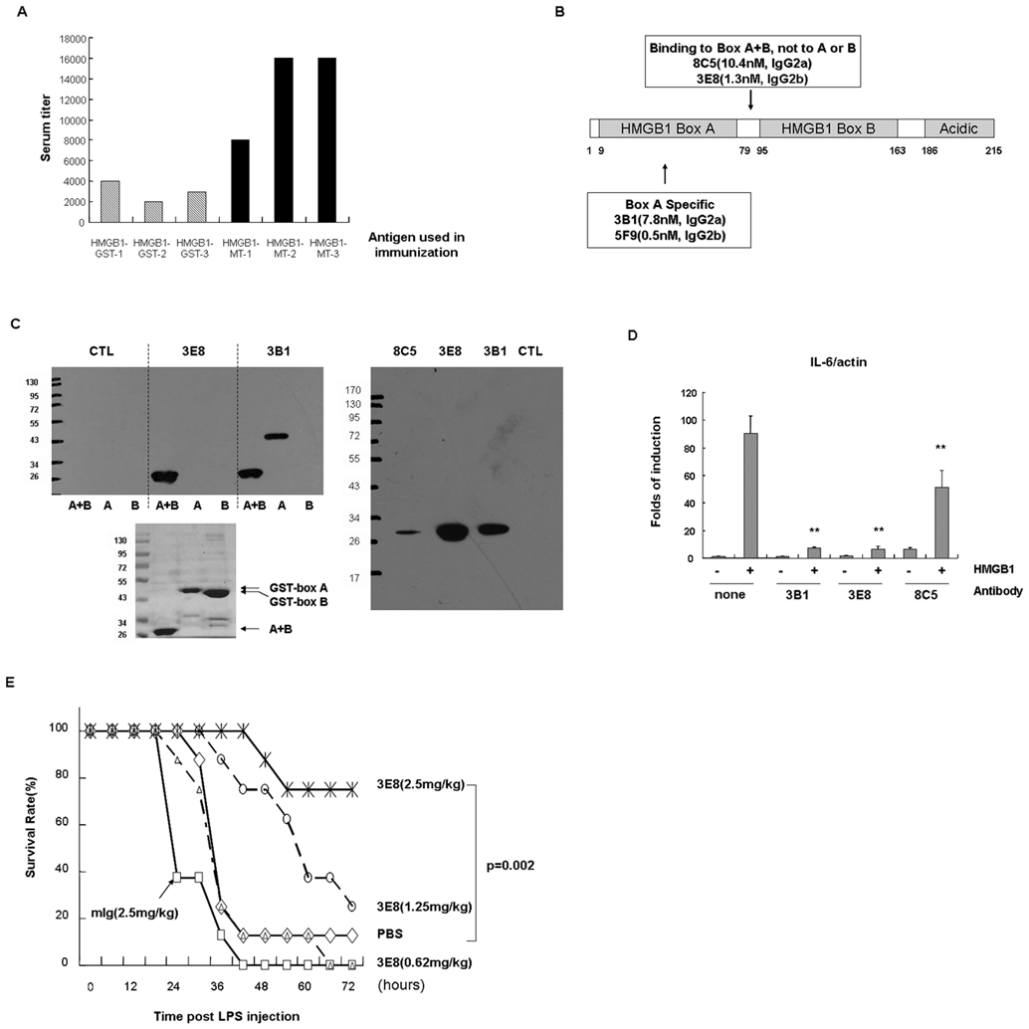
Generation of high affinity, biologically active anti-HMGB1 antibodies from NZB/W mice. (A) A T cell-specific tag fused to HMGB1 further increased immune response in NZB/W mice. NZB/W mice were immunized with GST-HMGB1 or HMGB1-MT. Serum titers were measured by ELISA using HMGB1-His-coated plates. Serum titer was defined as the highest dilution of serum at which the A450 ratio (A450 of sample/A450 of negative serum) was greater than 2.0. (B) Binding affinity and specificity of anti-HMGB1 antibodies.Antibody binding affinity was measured by ELISA using HMGB1-His-coated plates and the Kd of each antibody is indicated in the figure. Antibody specificity was measured by ELISA using the GST-HMGB1 Box (A), GST-HMGB1 Box (B) or GST-HMGB1 Box (A+B) as antigens. (C) Binding specificity of anti-HMGB-1 antibodies. Left panel: 0.3 µg of purified recombinant proteins (A+B:HMGB1-MT, A: GST-boxA, B:GST-boxB) were loaded and separated by electrophoresis on 10% SDS-PAGE gels. Western blots were carried out with 1% dry milk as the blocking buffer and 2 µg/ml purified antibodies as the primary antibody. The blots were then labeled with goat anti-mouse Ig-HRP and ECL substrate. 3 µg of purified recombinant proteins were separated by electrophoresis and stained with Coomassie Brilliant Blue for loading control. Right panel: 1×10^7^ Hela cells were lysed in 1 ml cell lysis buffer and mixed with 5× SDS sample buffer. 20 µl each of the above samples was separated by electrophoresis on 10% SDS-PAGE gels. Western blots were carried out with 5% BSA as the blocking buffer and 1 µg/ml biotinylated antibodies as the primary antibody. The blots were then labeled with streptavidin-HRP and ECL substrate. (D) anti-HMGB1 antibodies blocked HMGB1-induced IL-6 up-regulation. Raw264.7 cells were stimulated with 1 µg/ml HMGB1 for 8 hours, with or without 50 µg/ml of the antibodies indicated. Total mRNA was extracted with Trizol, and IL-6 mRNA levels were measured by real-time RT-PCR. Data are expressed as the means±SEM (n = 3). ** indicating p<0.01 when compared to samples treated with HMGB1 only. (E) Anti-HMGB1 mAb 3E8 protects mice from LPS-induced sepsis. 8 week-old C57BL/6 female mice, 8 mice/group, were injected intraperitoneally with 25 mg/kg LPS, along with three different doses of anti-HMGB1 antibody 3E8 or isotype control antibodies. The number of surviving mice was recorded every six hours.

HMGB1 is a proven therapeutic target in experimental models of ischemia, acute respiratory distress syndrome, rheumatoid arthritis, sepsis, and cancer [9], [15]. Due to the fact that the human and mouse HMGB1 proteins are highly conserved (98% identity) and that HMGB1-deficient mice die shortly after birth, it is hard to develop monoclonal antibodies against HMGB1. In this study, neither wild type nor NZB/W mice developed sufficient serum antibody titer when immunized with GST-tagged HMGB1 protein. We introduced a universal T cell epitope from a *Mycobacterium tuberculosis* antigen [16] into the C-terminus of HMGB1 and the resulting recombinant protein was used as an antigen for immunization. The T cell epitope greatly enhanced the immune response of NZB/W mice to HMGB1 (Fig. 2A). Out of 328 hybridoma clones screened, 17 hybridoma clones that secrete monoclonal antibodies specific for HMGB1 were identified. Four of these were high affinity clones, with Kds ranging from 0.5 nM to 10 nM (measured by ELISA). Two clones bound to the HMGB1 box A, the other two could only bind to the box A+B, failing to recognize the box A or box B (Fig. 2B). The binding specificities of clone 3B1 and 3E8 were further confirmed by western blot (Fig. 2C, left panel). When whole cell lysates were used, antibodies from clones 8C5, 3E8 and 3B1 specifically recognized the endogenous HMGB1in Hela cell lysates (Fig. 2C, right panel). Antibodies from the three clones also blocked HMGB1-induced IL-6 mRNA up-regulation in Raw264.7 cells (Fig. 2D), making these antibodies good candidates for therapeutic antibodies. In a LPS-induced sepsis model, one of the anti-HMGB1 clones, 3E8, was tested for *in vivo*-neutralizing activity. 3E8 protected mice effectively from lethal doses of LPS treatment (Fig. 2E). 3B1, a box A specific clone, had similar effects in protecting mice from LPS induced sepsis (data not shown).

**Figure 3:**
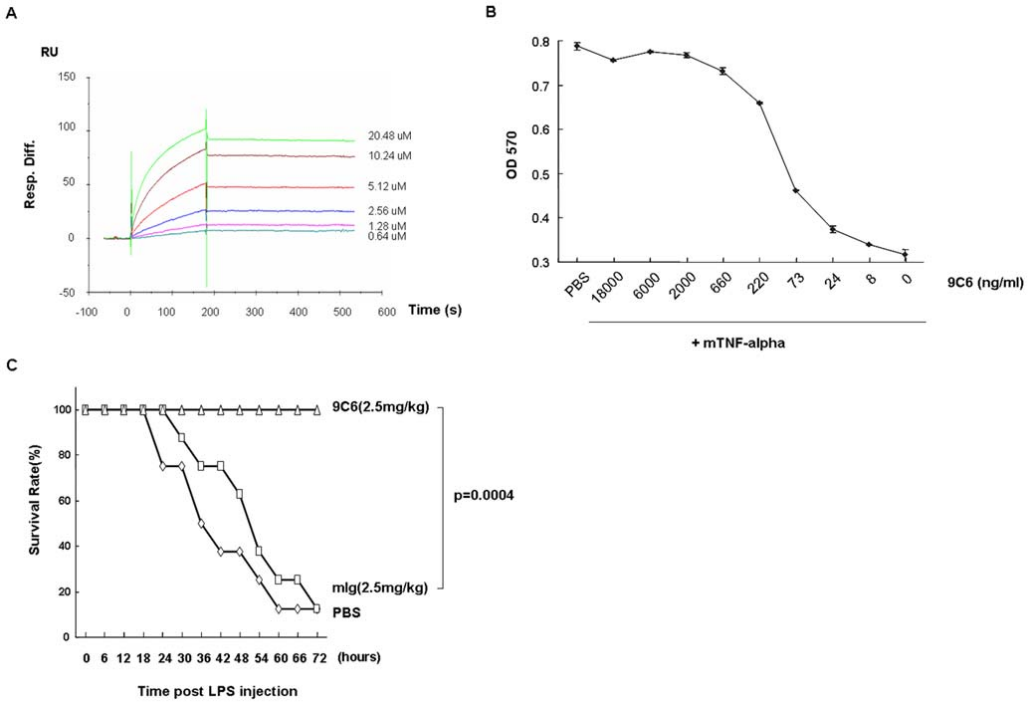
Characterization of anti-TNF-alpha antibody. (A) Binding affinity of anti-mouse-TNF-alpha antibody. The equilibrium constant of anti-mTNF-alpha antibody 9C6 was measured by Surface Plasmon Resonance (SPR) using a CM5 sensor chip in a BIACORE3000 at 25°C. Recombinant mouse-TNF-alpha was captured in the flow cell. Running buffer and different concentrations of anti-mTNFa mAb 9C6 (0.64, 1.28, 2.56, 5.12, 10.24, and 20.48 µM diluted in running buffer) were applied to the antigen-containing flow cell for 3 min at a flow rate of 30 µl/min. The Kd of 9C6 was calculated as 1.4 nM. (B) Anti-mTNF-alpha mAb 9C6 neutralizes TNF-alpha-induced cytotoxicity in L929 cells. 3.5×10^5^ L929 cells were treated with 1 µg/ml actinomycin D and 3 ng/ml mouse TNF-alpha for 15 hours. Different dilutions of anti-mTNFa mAb 9C6 (with a starting concentration of 18 µg/ml) were added to the assay. Cell death was measured by the MTT assay (Thiazolyl Blue Tetrazolium Bromide staining, read at 570 nm) [20]. (C) Anti-TNF-α mAb 9C6 protects mice from LPS-induced sepsis. 8 week-old C57BL/6 female mice, 8 mice/group, were injected intraperitoneally with 25 mg/kg LPS, along with 2.5 mg/kg anti-TNF-α mAb 9C6 or isotype control antibody. The number of surviving mice was recorded every six hours.

Generation of reagent antibodies suitable for *in vivo* use is essential in the target validation phase of antibody drug discovery projects. Rabbit polyclonal or even rat monoclonal antibodies will trigger an immune response in mice, and can render the antibody ineffective as early as the second week of treatment. In contrast, antibodies like mouse anti-mouse TNF-alpha are expected to avoid host responses and are desirable for efficacy assessments in mouse disease models and early safety studies. We immunized NZB/W mice with recombinant mouse TNF-alpha and successfully generated 20 hybridoma clones (out of 748 clones screened) secreting antibodies against mouse TNF-alpha. One antibody clone bound to mouse TNF-alpha with a high affinity (Kd = 1.4 nM, measured by Surface Plasmon Resonance on a Biacore3000, Fig. 3A), and good specificity (the epitope mapped to amino acids 19–40 of the TNF-alpha mature chain, data not shown), and could neutralize mouse TNF-alpha activity by blocking TNF-alpha's cytotoxic effect on L929 cells (Fig. 3B). It also showed good *in vivo* activity in the LPS-induced sepsis model (Fig. 3C).

## Discussion

Our work has demonstrated that immunization of NZB/W mice can be used as an effective and universal method to develop antibodies against self and highly conserved antigens.

The NZB/W mouse strain spontaneously develops antibodies against nucleic acids and self-nuclear proteins at 5–6 months of age. Using standard immunization protocols, we were able to induce strong antibody responses in this strain against highly conserved human antigen MIF or mouse self-antigen TNF-alpha before the onset of auto-immune disease. This is consistent with the notion that B cell tolerance is generally defective in NZB/W mice [17]. However, previous work has demonstrated that self-reactive T-cell cells assisting autoantibody production in NZB/W mice do not result from a generalized defect in T-cell tolerance, and that thymic and peripheral tolerance to most self-antigens are intact in NZB/W mice [18]. This could explain the weak antibody response observed here against HMGB1, a nuclear protein expressed at high level in all cells. To by-pass this obstacle, we introduced a strong T-cell epitope to the C terminus of recombinant HMGB1 protein. This T cell stimulatory epitope is from the 38-kDa lipoprotein of the *Mycobacterium tuberculosis*. Previous studies showed that this peptide can stimulate T cell proliferation in mice of H-2b, H-2d, and H-2k haplotypes [19]. Unlike regular fusion partners such as KLH, BSA or GST, this epitope does not elicit B cell response on its own, avoiding the disadvantages of autoreactive B cells in competing for T cell help [16]. Our data demonstrated that tagging the antigen with a T cell-specific epitope can further boost the immune response, and this response is stronger than that induced by a GST-HMGB1 fusion protein.

The NZB/W mouse strain has also been used to develop antibodies against bovine prion protein (PrP) [8]. Multiple hybridoma clones which secreted antibodies that bound specifically to different epitopes of PrP were isolated from NZB/W mice immunized with KLH-conjugated PrP. However, the binding affinity of these PrP-specific antibodies was not reported [8]. In our case when cytokines with clear therapeutic indications were chosen as test antigens, the goal was to develop high affinity antibodies with neutralization ability. We were able to demonstrate that antibodies generated with our method are mostly within the 1-10 nM Kd affinity range, sufficiently high for therapeutic purposes.
